# The influence of acetylsalicylic acid and alcohol on absorption kinetics of hen´s egg white in a human passive cutaneous anaphylaxis model

**DOI:** 10.29219/fnr.v65.7618

**Published:** 2021-11-25

**Authors:** Nicolaj Brandt, Esben Eller, Anja Pahlow Mose, Carsten Bindslev-Jensen, Charlotte Gotthard Mortz

**Affiliations:** Department of Dermatology and Allergy Center, Odense Research Center for Anaphylaxis (ORCA), Odense University Hospital, Odense C, Denmark

**Keywords:** Cofactors, augmentation factor, egg allergy, allergic reaction, adults, children

## Abstract

**Background:**

Despite the well-known fact that acetylsalicylic acid (ASA) can induce anaphylaxis in patients susceptible to wheat-dependent exercise-induced anaphylaxis, few studies have sought to investigate the effects of cofactors on type-1 food allergy and none with ASA and hen’s egg and hen’s egg and alcohol combined.

**Methods and results:**

We applied the experimental model of ‘passive cutaneous anaphylaxis’ in humans to study whether the absorption kinetics of egg white is altered while being treated with ASA or under the influence of alcohol. Donor sera from four egg allergic patients with specific immunoglobulin E (s-IgE) to ovalbumin (0.1–8.87–19.5–170 kUA/L) were injected intracutaneously into the forearm of 12 healthy volunteers who were then challenged separately to: 1) egg white 2) egg white + ASA and 3) egg white + alcohol. ‘Time to wheal’ and ‘wheal size’ were compared among the three experiments.

We saw that ‘time to wheal’ with both ASA (*P* = 0.001) and alcohol (*P* = 0.019) added as cofactor significantly decreased compared with baseline.

**Conclusion:**

In this passive cutaneous anaphylaxis model, ASA and alcohol affected both reaction time and size of reactions elicited after egg ingestion. This suggests that patients with egg allergy could have faster and more severe reactions during ASA treatment or under alcohol influence.

## Popular scientific summary

- This experimental model illustrates that the cofactors acetylsalicylic acid (ASA) and alcohol, also may exacerbate classical food allergy by giving a faster and more severe reaction. If this can be applied to the patient population, it means that the allergic patients can have a more severe reaction if exposed to a cofactor together with the allergen. This is an important information for the patients and for advising food allergic patients.

Cofactors or augmenting factors decrease the allergen dose required to elicit a reaction, that is, lowers the patient’s threshold and decreases the time to elicit a reaction, thereby putting allergic patients at risk of a more severe and potentially life-threatening anaphylactic reaction (1). Cofactors are best described in relation to wheat such as with wheat-dependent, exercise-induced anaphylaxis (WDEIA) (2), whereas data in relation to other allergens and different cofactors are sparse (3). This study aims to investigate the effect of acetylsalicylic acid (ASA) and alcohol in relation to hen’s egg allergy.

## Methods

For patients’ safety, the passive cutaneous anaphylaxis model (Prausnitz–Küstner) (4) was chosen. Briefly, serum from four egg-allergic patients containing specific IgE (s-IgE) to egg proteins were injected intracutaneously into the forearm of 12 healthy (11 male/1 female, median age 31 years [range 24–59]), non-allergic volunteers (recipients), thereby inducing a temporary sensitization in the primed areas. A subsequent oral challenge with egg white would then trigger a wheal-and-flare reaction at the injected sites, enabling measurement of absorption kinetics (4), that is, time to wheal formation (T_react_) and maximum size of the wheal (S_wheal_).

The day prior to each challenge, recipients were primed with 0.1 mL of each serum intracutaneously on the volar aspect of the forearm. The recipients avoided oral intake of egg for 72 h prior to serum transfer and abstained from food and fluid intake 8 h before the challenges. Details on the donor sera (DS1–DS4) are given in Table 1. All donors met the specific eligibility criteria outlined by the Danish Blood Safety Directives (5).

**Table 1:** Donor sera with threshold, challenge type and severity score with corresponding s-IgE (kUA/L)

	Donor serum 1	Donor serum 2	Donor serum 3	Donor serum 4
**Threshold egg**	300 mg (2006)	1,800 mg (2015)	800 mg (2007)	300 mg (2007)
**Challenge type**	Open	Open	Double-blind, placebo-controlled oral food challenge (DBPCFC)	DBPCFC
**Symptoms at challenge**	Oral allergy syndrome (OAS)	OAS, abdominal pain, rhino-conjunctivitis, sneezing, generalized urticaria, asthma	OAS, nausea, generalized urticaria, abdominal pain	OAS, abdominal pain, and repeated vomiting
**Severity score**	1	4	2	3
**S-IgE (kUA/L)**				
** Ovalbumin**	**0.1**	**8.87**	**19.5**	**170**
Ovomucoid	0.51	27.4	1.73	77.7
Lysozyme	<0.1	7.69	1.08	12.8
Conalbumin	<0.1	10.3	<0.1	52.5
Egg white	0.42	38	17.4	190
Egg yolk	<0.35	11.4	9.86	89.7
Total IgE	136	1,975	988	1,052

Note: Donor serum 2 was also peanut allergic with s-IgE to peanut at 665 kUA/L. Recipients should therefore additionally avoid peanut ingestion 3 days prior to priming. The threshold was determined using pasteurized whole egg measured in milligrams and the severity score was measured according to Sampson’s severity score. The s-IgE was sampled at the time of blood donation.

The recipients were challenged 24 h after priming with either: 1) 132 g of egg white (baseline), 2) 132 g of egg white with 1 g of ASA administered as two tablets of 500 mg Treo^R^ (Meda, Denmark) 30 min prior to egg white ingestion or 3) 132 g of egg white with the recipients’ blood alcohol level at 0.5 ‰ at challenge start, calculated from the recipients’ gender and weight and given 30 min before egg ingestion as commercially available vodka (37.5%). The blood alcohol level was measured hourly and maintained at 0.5‰. The primary endpoint was reaction time (T_react_), defined as time from ingestion of egg white to mean wheal diameter >3 mm. The secondary endpoint (S_wheal_) was maximal mean wheal diameter. Each experiment lasted 2 h. The effect of cofactors compared with baseline was evaluated by using the paired non-parametric Wilcoxon signed rank test. Significance level was set at *P* < 0.05. Calculations were performed using STATA^TM^ version 14.2 (StataCorp, USA).

## Results

**Fig. 1:**
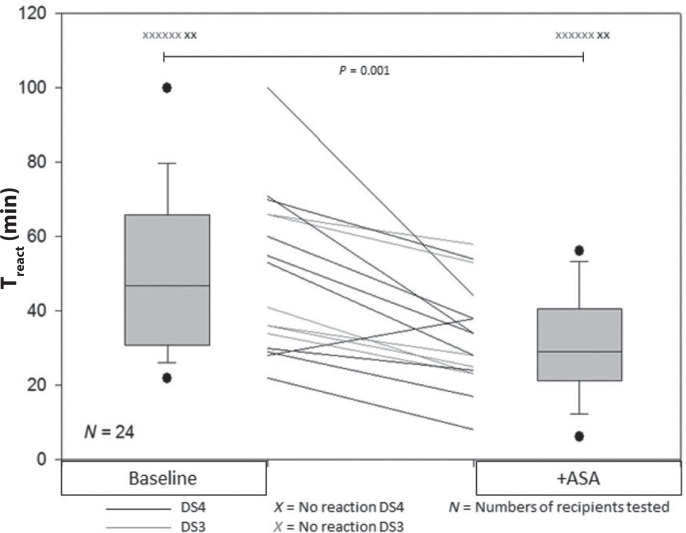
Reaction time without cofactors and with ASA. Pooled data from DS3 and DS4. Reaction time after ingestion of 132 g egg white measured as T_react_ without adding cofactors and with additional ingestion of ASA. The boxplots represent paired pooled data from DS3 and DS4 and each line represents one recipient. ASA, acetylsalicylic acid; DS#, Donor Serum number.

Reaction times for egg challenge without cofactors (baseline) and with addition of ASA or alcohol are presented in Fig. 1. Reactivity without cofactors (baseline) was correlated to donor IgE; none of the 12 recipients reacted with wheals to Donor Sera 1 (DS1) with the lowest s-IgE to ovalbumin and only one recipient to DS2. Donor Sera 1 and 2 were therefore excluded from further analysis. In case of DS3, six out of 12 recipients reacted with wheals, while 10 out of 12 recipients produced wheals with DS4 with the highest s-IgE to ovalbumin. Adding ASA as cofactor (Fig. 1) decreased the T_react_ for all six recipients producing a wheal with DS3 at baseline and nine out of the 10 recipients producing a wheal at baseline with DS4. The median T_react_ decreased from 47 min at baseline to 31 min with ASA. Reaction time was significantly lower in the pooled analysis of DS3 and DS4 (*P* = 0.001) comparing egg with ASA to egg alone.

**Fig. 2:**
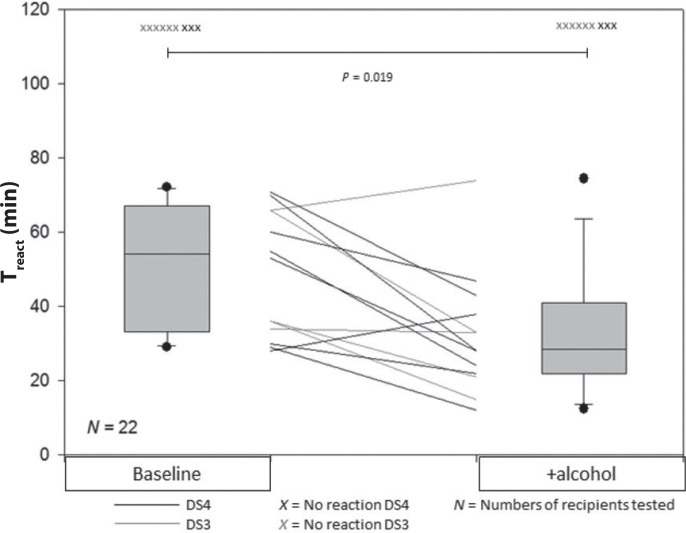
Reaction time without cofactors and with alcohol. Pooled data from DS3 and DS4. Reaction time after ingestion of 132 g egg white measured as T_react_ without adding cofactors (baseline) and with additional ingestion of alcohol. The boxplots represent paired pooled data from DS3 and DS4 and each line represents one recipient. DS#, Donor Serum number.

Reaction time with and without alcohol as cofactor is presented in Fig. 2. Only 11 recipients participated with alcohol as a cofactor and five of these reacted with a wheal on DS3 without a cofactor. Adding alcohol, four of the five recipients reacted faster and the remaining reacted slower. With DS4, eight of the 11 recipients reacted without cofactor and of these seven reacted faster with the addition of alcohol and one reacted slower. The median T_react_ decreased from 54 min without cofactor to 28 min with alcohol as cofactor. Overall, the reaction time was significantly faster with alcohol as cofactor, compared with egg white alone in a pooled analysis of DS3 and DS4 (*P* = 0.019).

Skin prick test (SPT) with whole egg was performed as positive control at the non-responding priming sites, eliciting a positive wheal at all sites with DS2, DS3 and DS4 whereas with DS1 a response was only seen in three of the 12 recipients, demonstrating that non-responders were not due to unsuccessful priming in the injected areas.

With DS3, a significant increase of 4.5 mm in Swheal was seen in five of the six recipients with addition of ASA (*P* = 0.046), whereas alcohol only increased wheal size non-significantly in three of the five recipients. With DS4, already producing maximal wheals at baseline, no significant increase was obtained.

## Discussion

Cofactors such as exercise, ASA and alcohol have been shown to influence the threshold of food allergy, primarily wheat allergy (1, 2). To investigate cofactors in egg allergy, we investigated absorption kinetics and dynamics of egg white with and without cofactors (ASA or alcohol) using sera from four egg allergic donors in 12 recipients in a passive cutaneous anaphylaxis model.

Reaction time as primary endpoint was significantly faster with the addition of ASA and alcohol. Reactivity was however correlated with donor IgE level, similar to recent findings for peanut (4); Both cofactors increased the wheal size S_wheal_ in the less potent IgE serum (DS3), whereas DS4 already resulted in maximum wheal size without cofactors. Our findings point toward cofactors being potentially involved in food allergy beyond food-dependent exercise-induced anaphylaxis (6). If our findings are reflected in the clinical situation, presence of cofactors could possibly augment the clinical reaction by lowering the threshold in the patient.

## Conclusion

We found that ASA and alcohol significantly affect the onset (time to reaction) and magnitude of local allergic reactions (wheal size). This indicates that egg allergic patients could have a faster and potentially more severe reaction when exposed to egg combined with ASA or alcohol intake.

## Authors’ contributions

NB, CGM and CBJ conceived of the presented idea. APM helped in the execution and planned the experiments. NB carried out the experiments and wrote the manuscript. EE and CGM verified the analytical methods. CBJ and CGM helped to supervise the project. All authors discussed the results and contributed to the final manuscript.

## Ethical statement

Permission from the Regional Danish Scientific Ethical Committee was obtained (Project-ID: S-20130086).

## Conflict of interest and funding

The Authors declare no conflicts of interest. Nicolaj Brandt received a one year scholarship from the Lundbeck foundation.

## References

[1] Wolbing F, Fischer J, Koberle M, Kaesler S, Biedermann T. About the role and underlying mechanisms of cofactors in anaphylaxis. Allergy 2013; 68(9): 1085–92. doi: 10.1111/all.12193 23909934

[2] Scherf KA, Brockow K, Biedermann T, Koehler P, Wieser H. Wheat-dependent exercise-induced anaphylaxis. Clin Exp Allergy 2016; 46(1): 10–20. doi: 10.1111/cea.12640 26381478

[3] Oropeza AR, Bindslev-Jensen C, Broesby-Olsen S, Kristensen T, Moller MB, Vestergaard H, et al. Patterns of anaphylaxis after diagnostic workup: A follow-up study of 226 patients with suspected anaphylaxis. Allergy 2017; 72(12): 1944–52. doi: 10.1111/all.13207 28543193

[4] Mose AP, Mortz CG, Eller E, Sprogoe U, Barington T, Bindslev-Jensen C. Dose-time-response relationship in peanut allergy using a human model of passive cutaneous anaphylaxis. J Allergy Clin Immunol 2017; 139(6): 2015–6.e4. doi: 10.1016/j.jaci.2016.11.034 28189335

[5] The Danish Blood Safety Directives (Bekendtgørelse nr 366 af 23/04/2012). Available from: https://www.retsinformation.dk/Forms/R0710.aspx?id=141589 [cited 8 July 2013].

[6] Niggemann B, Beyer K. Factors augmenting allergic reactions. Allergy 2014; 69(12): 1582–7. doi: 10.1111/all.12532 25306896

